# P-546. Eligibility and Factors Associated with Long-Acting Injectable Cabotegravir – Rilpivirine Initiation in a South Side Chicago HIV Clinic

**DOI:** 10.1093/ofid/ofae631.745

**Published:** 2025-01-29

**Authors:** Geoffroy Liegeon, Christopher Kaperak, Eleanor Friedman, Paul S Djuricich, Alicia Dawdani, Sophie Plotkin, Jessica Schmitt, Aniruddha Hazra, Katerina Christopoulos, John Schneider, Moira McNulty

**Affiliations:** University of Chicago, Chicago, Illinois; The University of Chicago, Chicago, Illinois; The University of Chicago, Chicago, Illinois; University of Chicago Medicine, Chicago, Illinois; University of Chicago, Chicago, Illinois; The University of Chicago, Chicago, Illinois; The University of Chicago, Chicago, Illinois; University of Chicago, Chicago, Illinois; University of California San Francisco, San Francisco, CA; University of Chicago Medicine, Chicago, Illinois; University of Chicago, Chicago, Illinois

## Abstract

**Background:**

We evaluated eligibility for long-acting injectable (LAI) cabotegravir (CAB) and rilpivirine (RPV) treatment among PWH in a Ryan White-funded HIV clinic and assessed factors associated with initiation to identify potential disparities in uptake.

**Methods:**

We conducted a retrospective cohort analysis among PWH who attended the University of Chicago HIV clinic from January 1, 2021 to May 31, 2023. PWH were considered eligible for LAI-CAB/RPV if they had HIV-1 RNA < 50 copies/mL for ≥3 months, no active hepatitis B virus infection, no resistance to rilpivirine or cabotegravir on historical genotypes, and/or no history of treatment failure on integrase inhibitors or non-nucleoside reverse transcriptase inhibitors. We used a logistic regression model to identify socio-demographic and clinical factors associated with treatment initiation among eligible PWH.

**Results:**

Of the 655 PWH, 413 (63%) were eligible for LAI-CAB/RPV. Median age was 45 years, median time from HIV diagnosis was 9 years, 33% were women, 84% Black, 70% had permanent housing, 52% were employed, 56% had Medicaid insurance, 9% had active substance use, 26% had an active mental illness, 69% were taking other daily oral medications, 13% were receiving another ongoing injectable treatment, 48% had no historical genotype available, and 6% had a CD4 count < 200/mm^3^. Of the 413 eligible PWH, 64 (15%) initiated LAI-CAB/RPV. PWH who initiated LAI-CAB/RPV were significantly younger (34 vs. 48 years, *P*< 0.001), diagnosed with HIV more recently (6 vs. 11 years, *P*=0.002), had a higher employment rate (67% vs 50%, *P*=0.03), and tended to have no mental health issues (19% vs. 28%, *P*=0.14). They were also less likely to take other daily pills (48% vs 73%, *P*< 0.001) but more likely to be receiving another injectable treatment (17% vs 12%, P=0.002). In multivariate analysis, younger age (*P*< 0.001) and not taking other daily pills (*P*=0.048) were associated with LAI CAB/RPV initiation.

**Conclusion:**

Younger age and not taking other daily pills were associated with LAI-CAB/RPV initiation. More work is needed to understand the reach of LAI-CAB/RPV for those eligible and interested in receiving it.

**Disclosures:**

**Eleanor Friedman, PhD**, Gilead health sciences: Grant/Research Support **Aniruddha Hazra, MD**, Gilead Sciences: Advisor/Consultant|Gilead Sciences: Grant/Research Support|ViiV Healthcare: Advisor/Consultant **Katerina Christopoulos, MD, MPH**, Janssen: Honoraria **Moira McNulty, MD**, Gilead Sciences, Inc.: Advisor/Consultant

